# Prediction of tumor lysis syndrome in childhood acute lymphoblastic leukemia based on machine learning models: a retrospective study

**DOI:** 10.3389/fonc.2024.1337295

**Published:** 2024-03-07

**Authors:** Yao Xiao, Li Xiao, Yang Zhang, Ximing Xu, Xianmin Guan, Yuxia Guo, Yali Shen, XiaoYing Lei, Ying Dou, Jie Yu

**Affiliations:** ^1^ Department of Hematology and Oncology, Children’s Hospital of Chongqing Medical University, National Clinical Research Center for Child Health and Disorders, Chongqing Key Laboratory of Pediatrics, Ministry of Education Key Laboratory of Child Development and Disorders, Chongqing, China; ^2^ College of Medical Informatics, Chongqing Medical University, Chongqing, China; ^3^ Big Data Engineering Center for Children’s Medical Care, Children’s Hospital of Chongqing Medical University, Chongqing, China

**Keywords:** machine learning, predictive modeling, acute lymphoblastic leukemia, tumor lysis syndrome, treatment toxicity

## Abstract

**Background:**

Tumor lysis syndrome (TLS) often occurs early after induction chemotherapy for acute lymphoblastic leukemia (ALL) and can rapidly progress. This study aimed to construct a machine learning model to predict the risk of TLS using clinical indicators at the time of ALL diagnosis.

**Methods:**

This observational cohort study was conducted at the National Clinical Research Center for Child Health and Disease. Data were collected from pediatric ALL patients diagnosed between December 2008 and December 2021. Four machine learning models were constructed using the Least Absolute Shrinkage and Selection Operator (LASSO) to select key clinical indicators for model construction.

**Results:**

The study included 2,243 pediatric ALL patients, and the occurrence of TLS was 8.87%. A total of 33 indicators with missing values ≤30% were collected, and 12 risk factors were selected through LASSO regression analysis. The CatBoost model with the best performance after feature screening was selected to predict the TLS of ALL patients. The CatBoost model had an AUC of 0.832 and an accuracy of 0.758. The risk factors most associated with TLS were the absence of potassium, phosphorus, aspartate transaminase (AST), white blood cell count (WBC), and urea levels.

**Conclusion:**

We developed the first TLS prediction model for pediatric ALL to assist clinicians in risk stratification at diagnosis and in developing personalized treatment protocols. This study is registered on the China Clinical Trials Registry platform (ChiCTR2200060616).

**Clinical trial registration:**

https://www.chictr.org.cn/, identifier ChiCTR2200060616.

## 1 Introduction

Acute lymphoblastic leukemia (ALL) is a malignant tumor of the hematopoietic system caused by the abnormal proliferation of bone marrow T- or B-lineage lymphocytes, and it is the most common malignant disease in children, accounting for about one-third of all childhood malignancies (1). The incidence rate of ALL is about (3~5)/100,000 (2), and the age of onset is mostly before 15 years old (3, 4). The male-to-female ratio is about 1.2:1 (5). In recent years, chemotherapy based on risk factor stratification has significantly improved the prognosis of children with ALL. In developed countries, the 5-year event-free survival (EFS) of pediatric ALL can reach more than 85%, and the overall survival (OS) can reach more than 90% (1, 6, 7). In China, the 5-year EFS of pediatric ALL reaches 80%, and the OS reaches more than 85% (8). However, about 20% of ALL patients face treatment failure, mainly due to recurrence, secondary tumors, chemotherapy toxicity, or severe complications (9). Several studies have reported that 30% to 47% of ALL patients die from treatment-related deaths (8, 10, 11), with infection and bleeding being the main causes. Among these serious complications, tumor lysis syndrome (TLS) often occurs early after induction chemotherapy in patients with ALL, and most of them progress rapidly. Acute kidney injury (AKI), cardiac arrhythmia, seizures, and even death may occur at the time of diagnosis. An in-depth understanding of TLS is beneficial to optimize the efficacy of ALL and provide a reference for TLS prevention and treatment.

TLS is an oncologic emergency caused by the massive lysis of tumor cells and the release of large amounts of potassium, phosphate, and nucleic acids into the systemic circulation. Clinical manifestations are characterized by hyperuricemia, hyperkalemia, hyperphosphatemia, and hypocalcemia (12, 13). Standard treatment for TLS includes massive hydration, allopurinol, and labyrinthine for hyperuricemia, and renal replacement therapy may be required in severe cases (14) Clinical observations suggest that TLS tends to occur in highly proliferative malignancies with heavy tumor loads or sensitive responses to initial therapy, such as Burkitt’s lymphoma and ALL (15). Thus, TLS is a great challenge in pediatric ALL. The analysis of independent risk factors for the development of TLS in children with ALL in the early stage of induction chemotherapy based on laboratory tests and clinical presentation at the time of initial diagnosis and the construction of a predictive model are crucial for the timely adoption of stronger interventions and close monitoring of high-risk patients to improve patient survival.

Currently, machine learning is widely used in the fields of disease diagnosis, prediction of prognosis, design of treatment plans, and individualized healthcare (16). Previous studies have reported the prediction models for the occurrence of TLS in AML in adults and ALL in children abroad (17–19). However, machine learning-based TLS prediction models for childhood ALL have not been reported at home or abroad. Relying on the clinical research big data platform of the National Clinical Medical Research Center for Children’s Health and Diseases, this study intends to construct a machine learning model to predict the probability of TLS in ALL patients by analyzing the clinical characteristics of real-world patients with ALL to provide a basis for decision-making for the early identification and intervention of TLS.

## 2 Materials and methods

### 2.1 Study population and design

This observational retrospective cohort study was conducted at the Children’s Hospital of Chongqing Medical University, one of two national clinical research centers for child health and disease in China that completed the Clinical Science Research Big Data Platform (CSRBDP) in 2021, which is available to clinical researchers and includes more than 750,000 pediatric outpatients and inpatients as of January 2022. To establish and validate the prediction model, the subjects were randomly divided into a training set (*n* = 1,570) and a validation set (*n* = 673) in a 7:3 ratio. The inclusion criteria were as follows: (1) age between 0 and 18 years; (2) diagnosis made between December 2008 and December 2021; (3) newly diagnosed ALL patients or out-of-hospital-diagnosed patients who did not receive steroids and whose bone marrow smear showed a ratio of primitive/naïve lymphocytes ≥20%. The exclusion criteria were as follows: (1) patients with secondary tumors or immunodeficiency diseases and (2) patients who did not have uric acid (UA), potassium, phosphorus, and calcium indexes detected within the 3 days before and 7 days after baseline time, where the baseline time was defined as the time of the first steroid administration at the time of hospitalization.

**Figure 1:**
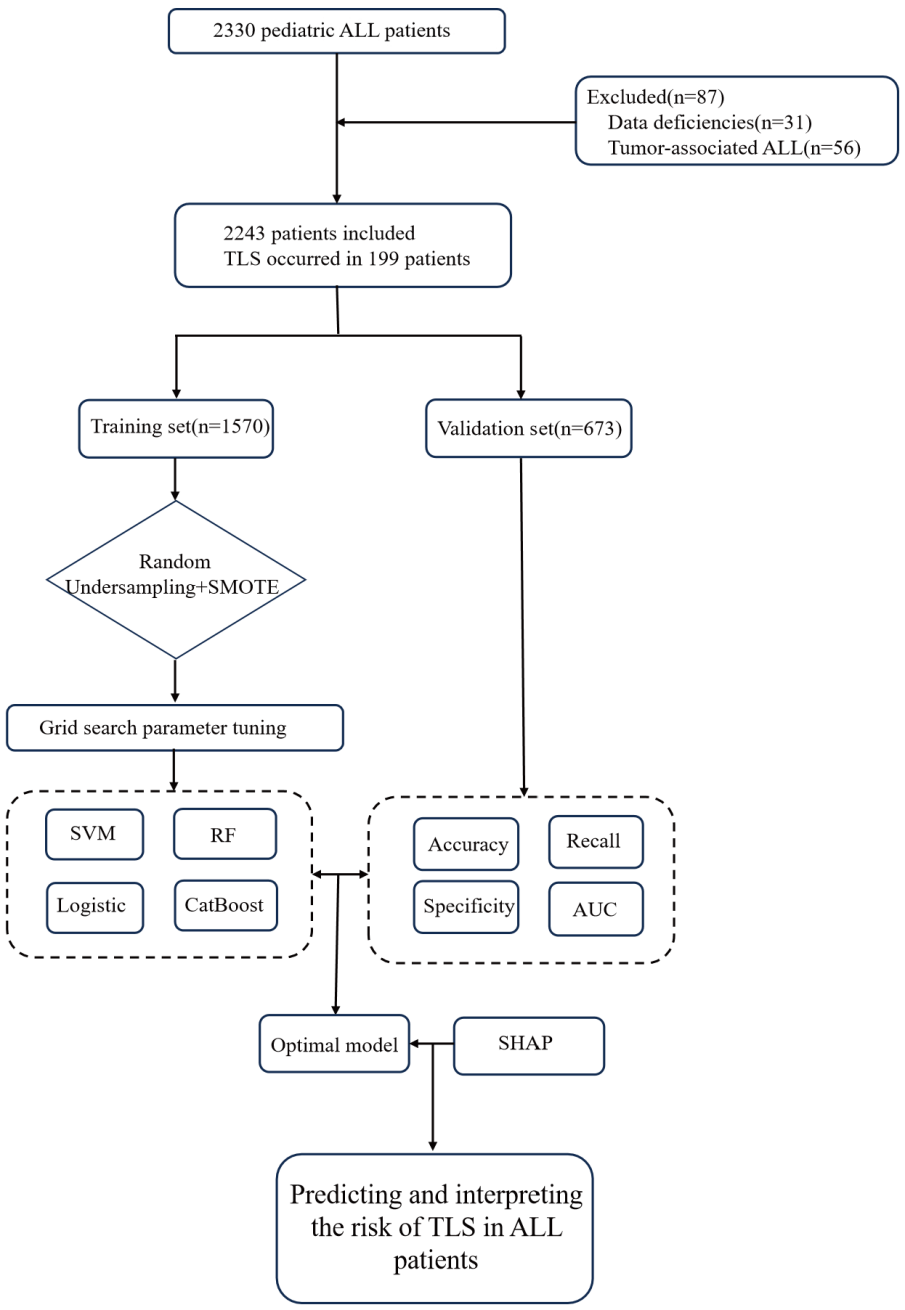
Machine learning model construction process.

The study followed the Transparent Reporting of Individual Prognostic or Diagnostic Multivariate Predictive Models (TRIPOD) specifications for model development and validation (20). Multidimensional clinical data were collected from the CSRBDP, and the study outcome was whether TLS occurred after chemotherapy. The study was registered on the Chinese clinical trial registration platform (clinicaltrials.gov, identifier: ChiCTR2200060616) and was approved by the Ethics Committee of the Children’s Hospital of Chongqing Medical University with a waiver of the informed consent requirement (File No. 2022,98). **Figure 1** presents an overview of the study design.

### 2.2 Data collection

A total of 33 clinically relevant indicators of ALL were collected in this study, including demographic information (e.g., age at diagnosis, gender), laboratory indicators (e.g., blood counts, liver and kidney function, electrolytes, and coagulation), and the type of steroids used at the time of the initial induction chemotherapy. Clinical and laboratory data were collected from all visits, starting with the patient’s initial diagnosis of ALL. In cases where this information was not available, data collected during the patient’s hospital stay were used instead.

### 2.3 Chemotherapy regimen

The chemotherapy regimen is based on international and national treatment protocols for childhood ALL (21–23). It stratifies chemotherapy according to the risk level (low risk, intermediate risk, and high risk). The main chemotherapeutic regimens include window-phase treatment, VDLP(Pred+VCR+DNR+L-ASP/PEG-ASP) induction chemotherapy, CAM(CTX+Ara-C+6-MP) early intensive therapy, high-dose MTX (HDMTX) consolidation therapy, reinduction therapy, and maintenance chemotherapy. TLS mainly occurs during the window-phase treatment and induction of remission therapy phases of the initial treatment. The main drugs used in remission-induction therapy include steroids (Pred/Dex), VCR, DNR, L-ASP/PEG-ASP, and intrathecal injections (steroids, MTX, Ara-C). BCR-ABL1-positive children are given chemotherapy combined with tyrosine kinase inhibitors (TKI).

### 2.4 Prevention of TLS

Routine preventive measures for TLS include the following: (1) Hydration therapy: adequate hydration therapy and maintaining the balance of fluid and output is the most important measure in the prevention and treatment of TLS (24). The recommended dose of hydration solution for children is 2,000~3,000 mL/(m^2^/day), and for infants with a body mass less than 10 kg, it is 200 mL/(kg/day) (25). Infants should maintain a urine output of more than 4 mL/(kg/h), while children (excluding infants) should have a urine output of more than 100 mL/(m^2^/h) to ensure proper renal perfusion and excretion of related metabolites (19). Hourly monitoring of urine output is essential for hydrated children to detect and treat circulatory overload promptly to avoid circulatory failure. If urine output is insufficient, non-thiazide diuretics (such as furosemide 0.5–1.0 mg/kg) can be used for diuresis, although they may impair renal function and promote the deposition of uric acid in the kidneys (26). (2) Correction of hyperuricemia: treatment options for hyperuricemia include xanthine oxidase inhibitors (such as allopurinol) and uric acid oxidase (such as acrylate). Allopurinol may be the initial drug of choice for low-risk TLS and intermediate-risk patients with normal serum uric acid levels. In the absence of high serum uric acid levels, prophylactic treatment with allopurinol is recommended for patients at intermediate risk for TLS, starting 24–48 h before chemotherapy initiation with a routine oral dosage of 10 mg/(kg/day) in three divided doses (the maximum daily dose should not exceed 800 mg). Meanwhile, diuretics should be discontinued, and the dosage of drugs such as cyclosporine should be adjusted. Patients with G-6-PD negative can be given uric acid oxidase at a dosage of 1.5 mg every other day until WBC is less than 50 × 10^9^/L, LDH is within two times the normal upper limit, and UA levels are normal. (3)Symptomatic support: correcting electrolyte disturbances and hemodialysis in emergencies can be used to treat oliguric renal failure or life-threatening metabolic disorders.

### 2.5 Definition of TLS

The recent international guidelines for the diagnosis of TLS include the ASCO guidelines of 2008 (27), the 2010 International Expert Consensus (25), and the 2015 BCSH guidelines (28). These guidelines primarily apply to adults. In 2021, the Pediatric Tumor Committee of the Chinese Anti-Cancer Association published guidelines for the diagnosis and treatment of TLS in Chinese children. The 2004 Cairo–Bishop definition has been widely adopted by international guidelines (29). According to the Cairo–Bishop criteria, laboratory TLS (LTLS) is diagnosed when there is a specified absolute or 25% increase/decrease from baseline in serum levels of UA, potassium, phosphorus, and/or calcium. The specified absolute serum levels are as follows: uric acid, ≥8 mg/dL; potassium, ≥6 mmol/L; phosphorus, ≥6.5 mg/dL in children and ≥4.5 mg/dL in adults; and calcium, ≤7 mg/dL. TLS is diagnosed when two or more of the proposed criteria are met, either 3 days before or 7 days after chemotherapy administration, regardless of hydration status and the use of hyperuricemic agents. Clinical TLS (CTLS) is defined as the presence of LTLS and at least one complication associated with TLS, without any other identifiable cause, such as an increase in serum creatinine (Crea) of ≥1.5 times the upper limit of normal (ULN), cardiac arrhythmia, sudden death, hand and foot cramps, or seizures.

### 2.6 Model development

In this study, indicators with missing rates >30% were excluded, and for indicators with missing rates ≤30%, the Random Forest-based iterative interpolation method (Miss Forest) (30) was used to fill in the missing values. The Least Absolute Shrinkage and Selection Operator (LASSO) was applied to identify influential clinical variables (predictor variables with *p* < 0.05) to remove irrelevant and redundant information, select the best predictor variables, and improve the predictive power of machine learning models.

Random stratified sampling divided the patients into training and validation sets in a ratio of 7:3, with the former being used to train the machine learning models and the latter being used to evaluate the model performance. Four machine learning models were constructed based on the training set, including Support Vector Machine (SVM), Random Forest (RF), CatBoost Classifier (CatBoost), and Logistic Regression (Logistic). The training set was preprocessed using random undersampling combined with the SMOTE oversampling technique to deal with sample imbalance. Hyperparameter tuning of machine learning models uses fivefold cross-validation grid search, and model construction in the training set uses fivefold cross-validation (31). The best-performing model was chosen for risk prediction based on its combined metrics of AUC, recall, specificity, and accuracy. The predictive power of the best machine learning models was explained using Shapley additive explanations (SHAP) (32).

### 2.7 Statistical methods

Statistical analyses were performed using R (version 4.3.0), while predictive model construction and evaluation were conducted using Python (version 3.7). Continuous variables were assessed for normality using the Shapiro–Wilk test, and nonnormal continuous variables were presented as median with interquartile range (IQR). Categorical variables were presented as frequencies and percentages (*n*, %). The Mann–Whitney *U*-test was used for continuous variables, and the Chi-square test was used for categorical variables to compare the differences in variable distribution between the training and validation cohorts. Statistical significance was defined as a two-sided *p*-value < 0.05.

## 3 Results

### 3.1 General patient characteristics

A total of 2,243 patients diagnosed with ALL were included in this study, including 1,332 male (59%) and 911 female (41%) patients, with an incidence of TLS of 8.87% (199/2243). A total of 33 metrics were analyzed after excluding metrics with missing values >30%. **Table 1** presents a comparison of clinical and laboratory characteristics between patients in the training and validation cohorts at diagnosis. The results indicate that there were no significant differences between the two cohorts (*p* > 0.05). We conducted a comparison of the basic characteristics of patients with or without the TLS. The analysis revealed that gender, immunophenotypes, French–American–British classification systems (FAB), white blood cell count (WBC), creatinine (Crea), urea, phosphorus, potassium, uric acid (UA), lactate dehydrogenase (LDH), aspartate transaminase (AST), alanine transaminase (ALT), total bilirubin (TBil), fibrinogen (Fib), prothrombin time (PT), primitive immature cells, the occurrence of hemorrhage, cardiac arrhythmia, AKI, and splenomegaly had a statistically significant relationship with the occurrence of TLS (*p* < 0.05), as shown in **Supplementary Table S1**.

**Table 1:** Comparison of demographic and clinical indicators in the training cohort and validation cohort of patients with ALL.

Characteristic	Group			P-value
	Overall, N = 2,243	Trainset, N= 1,570	Testset, N = 673	
**TLS, n(%)**				0.661
No	2,044 (91%)	1,428 (91%)	616 (92%)	
Yes	199 (8.9%)	142 (9.0%)	57 (8.5%)	
**Gender, n(%)**				0.472
Male	1,332 (59%)	940 (60%)	392 (58%)	
Female	911 (41%)	630 (40%)	281 (42%)	
**Age, n(%)**				0.491
<1 years	47 (2.1%)	30 (1.9%)	17 (2.5%)	
1~10 years	1,808 (81%)	1,274 (81%)	534 (79%)	
≥10 years	388 (17%)	266 (17%)	122 (18%)	
**immunophenotype, n (%)**				0.188
Common B-cell	2,041 (91%)	1,422 (91%)	619 (92%)	
Precursor B-cell	107 (4.8%)	73 (4.6%)	34 (5.1%)	
T-cell	89 (4.0%)	71 (4.5%)	18 (2.7%)	
Other (biphenotypic)	6 (0.3%)	4 (0.3%)	2 (0.3%)	
**FAB, n (%)**				0.489
L1	830 (37%)	595 (38%)	235 (35%)	
L2	1,191 (53%)	817 (52%)	374 (56%)	
L3	148 (6.6%)	106 (6.8%)	42 (6.2%)	
Unclassified	74 (3.3%)	52 (3.3%)	22 (3.3%)	
**regimen, n (%)**				0.800
CCCG-2008	758 (34%)	525 (33%)	233 (35%)	
CCCG-2015	1,040 (46%)	735 (47%)	305 (45%)	
CCCG-2020	445 (20%)	310 (20%)	135 (20%)	
**WBC, n (%)**				0.927
<50 x10^9^/L	1,876 (84%)	1,310 (83%)	566 (84%)	
50~100 x10^9^/L	165 (7.4%)	117 (7.5%)	48 (7.1%)	
≥100 x10^9^/L	202 (9.0%)	143 (9.1%)	59 (8.8%)	
**PLT, n (%)**				0.864
<100 x10^9^/L	1,524 (68%)	1,065 (68%)	459 (68%)	
≥100 x10^9^/L	719 (32%)	505 (32%)	214 (32%)	
**Hb, n (%)**				0.638
<30 g/L	9 (0.4%)	7 (0.4%)	2 (0.3%)	
30~60 g/L	255 (11%)	170 (11%)	85 (13%)	
60~90 g/L	1,208 (54%)	849 (54%)	359 (53%)	
90~120 g/L	619 (28%)	432 (28%)	187 (28%)	
≥120 g/L	152 (6.8%)	112 (7.1%)	40 (5.9%)	
**Cr, n (%)**				0.393
<69.7 umol/L	2,155 (96%)	1,512 (96%)	643 (96%)	
≥69.7 umol/L	88 (3.9%)	58 (3.7%)	30 (4.5%)	
**Urea, n (%)**				0.457
<6.5 mmol/L	2,003 (89%)	1,407 (90%)	596 (89%)	
≥6.5 mmol/L	240 (11%)	163 (10%)	77 (11%)	
**Ca, n (%)**				>0.999
<1.12 mmol/L	2 (<0.1%)	2 (0.1%)	0 (0%)	
≥1.12 mmol/L	2,241 (100%)	1,568 (100%)	673 (100%)	
**P, n (%)**				0.855
<2.1 mmol/L	2,147 (96%)	1,502 (96%)	645 (96%)	
≥2.1 mmol/L	96 (4.3%)	68 (4.3%)	28 (4.2%)	
**K, n (%)**				0.410
<3.5 mmol/L	201 (9.0%)	144 (9.2%)	57 (8.5%)	
3.5~5.5 mmol/L	2,028 (90%)	1,414 (90%)	614 (91%)	
≥5.5 mmol/L	14 (0.6%)	12 (0.8%)	2 (0.3%)	
**Uric, n (%)**				0.960
<476 umol/L	1,825 (81%)	1,277 (81%)	548 (81%)	
≥476 umol/L	418 (19%)	293 (19%)	125 (19%)	
**LDH, n (%)**				0.323
<245 U/L	469 (21%)	337 (21%)	132 (20%)	
≥245 U/L	1,774 (79%)	1,233 (79%)	541 (80%)	
**AST, n (%)**				0.400
<50 U/L	1,609 (72%)	1,118 (71%)	491 (73%)	
≥50 U/L	634 (28%)	452 (29%)	182 (27%)	
**ALT, n (%)**				0.396
<40 U/L	1,768 (79%)	1,230 (78%)	538 (80%)	
≥40 U/L	475 (21%)	340 (22%)	135 (20%)	
**TBil, n (%)**				0.276
<17.1 umol/L	2,104 (94%)	1,467 (93%)	637 (95%)	
≥17.1 umol/L	139 (6.2%)	103 (6.6%)	36 (5.3%)	
**Fib, n (%)**				0.378
<2 g/L	510 (23%)	365 (23%)	145 (22%)	
≥2 g/L	1,733 (77%)	1,205 (77%)	528 (78%)	
**Blasts, n (%)**				0.487
<5%	2,203 (98%)	1,544 (98%)	659 (98%)	
≥5%	40 (1.8%)	26 (1.7%)	14 (2.1%)	
**glucose, n (%)**				0.862
<6.1 mmol/L	1,818 (81%)	1,274 (81%)	544 (81%)	
≥6.1 mmol/L	425 (19%)	296 (19%)	129 (19%)	
**PT, n (%)**				0.168
<13秒	1,632 (73%)	1,129 (72%)	503 (75%)	
≥13秒	611 (27%)	441 (28%)	170 (25%)	
**CRP, n (%)**				0.249
<8 mg/L	244 (11%)	163 (10%)	81 (12%)	
≥8 mg/L	1,999 (89%)	1,407 (90%)	592 (88%)	
**hemorrhage, n (%)**				0.654
No	1,217 (54%)	847 (54%)	370 (55%)	
Yes	1,026 (46%)	723 (46%)	303 (45%)	
**epilepsy, n (%)**				0.298
No	2,233 (100%)	1,561 (99%)	672 (100%)	
Yes	10 (0.4%)	9 (0.6%)	1 (0.1%)	
**convulsions, n (%)**				>0.999
No	2,230 (99%)	1,561 (99%)	669 (99%)	
Yes	13 (0.6%)	9 (0.6%)	4 (0.6%)	
**infection, n (%)**				0.287
No	157 (7.0%)	104 (6.6%)	53 (7.9%)	
Yes	2,086 (93%)	1,466 (93%)	620 (92%)	
**HF, n (%)**				0.733
No	2,233 (100%)	1,562 (99%)	671 (100%)	
Yes	10 (0.4%)	8 (0.5%)	2 (0.3%)	
**AKI, n (%)**				0.704
No	2,216 (99%)	1,552 (99%)	664 (99%)	
Yes	27 (1.2%)	18 (1.1%)	9 (1.3%)	
**arrhythmia, n (%)**				0.206
No	1,595 (71%)	1,104 (70%)	491 (73%)	
Yes	648 (29%)	466 (30%)	182 (27%)	
**hepatomegaly, n (%)**				0.301
No	476 (21%)	324 (21%)	152 (23%)	
Yes	1,767 (79%)	1,246 (79%)	521 (77%)	
**splenomegaly, n (%)**				0.081
No	1,027 (46%)	700 (45%)	327 (49%)	
Yes	1,216 (54%)	870 (55%)	346 (51%)	
**Steroid, n (%)**				0.820
Pred	483 (22%)	337 (21%)	146 (22%)	
Dex	1,614 (72%)	1,134 (72%)	480 (71%)	
Unknown	146 (6.5%)	99 (6.3%)	47 (7.0%)	

TLS, tumor lysis syndrome; FAB, French–American–British classification systems; WBC, white blood cell count; Hb, hemoglobin; P, phosphorus; K, potassium; Uric, uric acid; Cr, creatinine; LDH, lactate dehydrogenase; AST, aspartate transaminase; ALT, alanine transaminase; TBil, total bilirubin; Fib, fibrinogen; Blasts, primitive immature cells; PT, prothrombin time; CRP, C-reactive protein; HF, heart failure; AKI, acute kidney injury; Pred, prednisone; Dex, dexamethasone.

### 3.2 Feature screening

**Figure 2:**
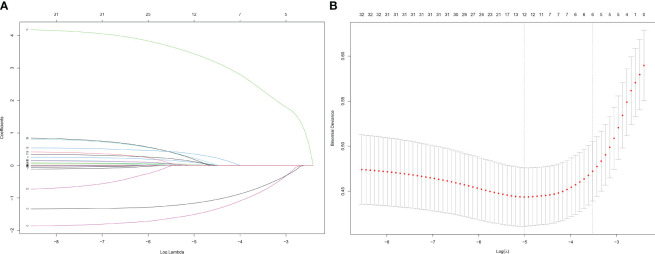
Screening of variables based on Lasso regression. **(A)** The variation characteristics of the coefficient of variables; **(B)** the selection process of the optimum value of the parameter λ in the Lasso regression model by cross-validation method.

In the LASSO regression for feature screening, 33 candidate predictor variables were included. By adjusting *λ*, the number of variables was reduced, which resulted in a simplified model. The number of clinical characteristics of pediatric ALL patients decreased continuously with increasing log (*λ*) (**Figure 2A**). By performing 10-fold cross-validation to find the optimal *λ* value, LASSO selected 12 variables with nonzero regression coefficients at *λ* = 0.007 and log (*λ*) = −2.176 (**Figure 2B**). The 12 predictor variables that were ultimately included in the follow-up study were FAB type, WBC, phosphorus, calcium, potassium, UA, AST, blood glucose, occurrence of infection, AKI, cardiac arrhythmia, and the type of steroid used in initial induction chemotherapy.

### 3.3 Model development and screening

**Figure 3:**
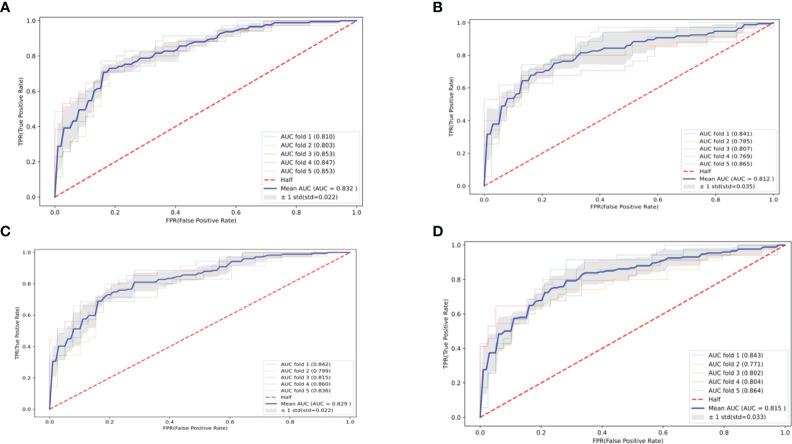
Four machine learning-based predictive model ROC curves after feature screening. **(A)** CatBoost; **(B)** Logistic; **(C)** Random Forest; **(D)** SVM.

**Figure 4:**
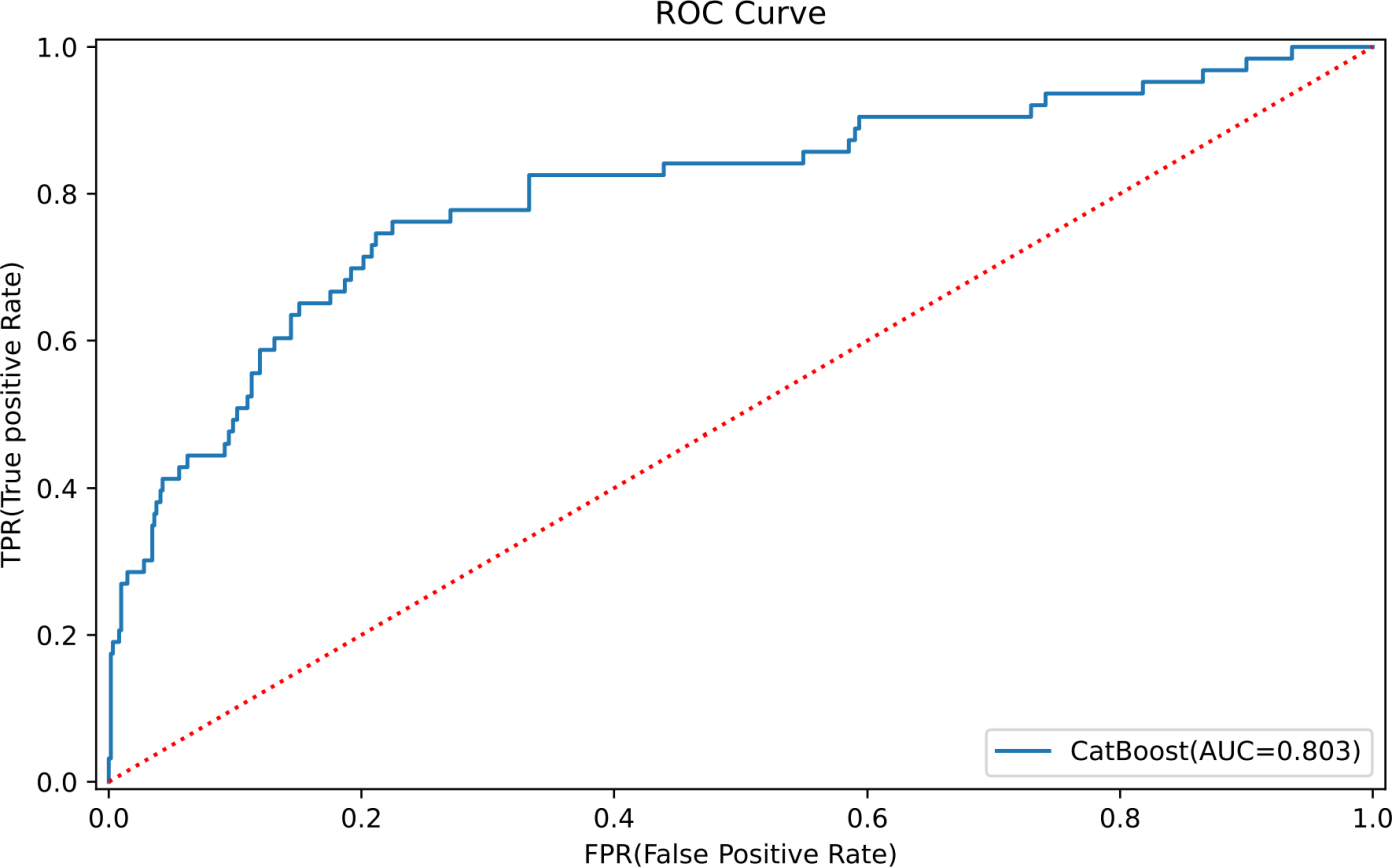
The optimal model, CatBoost ROC curves, in the validation set.

The optimal parameters for developing the TLS prediction model for ALL in children through grid search and cross-validation are presented in **Supplementary Table S2**. Four postscreening prediction models that included 12 variables using LASSO regression feature screening were included. Among the four predictive models constructed after LASSO regression screening, the CatBoost model has the best prediction with AUC = 0.832 (95% CI: 0.810–0.854) (**Figure 3A**), while the Logistic model has AUC = 0.812 (95% CI: 0.777–0.847) (**Figure 3B**); the AUC = 0.829 (95% CI: 0.807–0.851) for the Random Forest model (**Figure 3C**); AUC = 0.815 (95% CI: 0.782–0.848) for the SVM model (**Figure 3D**). To comprehensively evaluate the prediction performance of each model, the accuracy, specificity, recall, and Brier scores of the four models were also calculated in this study. **Table 2** shows the performance parameters of the four models constructed after LASSO feature screening. The CatBoost model has an accuracy of 0.758, a recall of 0.684, and a specificity of 0.825. After the screening of the LASSO regression features, we finally selected the CatBoost model as the optimal model, and the optimal model, CatBoost, was evaluated in the validation set, with an AUC of 0.803 (95% CI: 0.735–0.865, accuracy: 0.807, recall: 0.667, specificity: 0.821(**Figure 4**).

**Table 2:** Model performance (after feature screening).

Model	AUC (95% confidence interval)	Accuracy	Recall rate	Idiosyncrasy	Brier
CatBoost	0.832 (0.810–0.854)	0.758	0.684	0.825	0.168
Random Forest	0.829 (0.807–0.851)	0.769	0.707	0.824	0.170
SVM	0.815 (0.782–0.848)	0.739	0.736	0.742	0.175
Logistic	0.812 (0.777–0.847)	0.747	0.650	0.835	0.175

### 3.4 Interpretation of personalized predictions

To visually explain the selected variables, this study used SHAP to illustrate how these variables affect TLS in the model. The feature importance ranking of the CatBoost is shown in **Supplementary Figure S1**. The factors most associated with TLS were potassium, phosphorus, AST, WBC, and UA.

**Figure 5:**
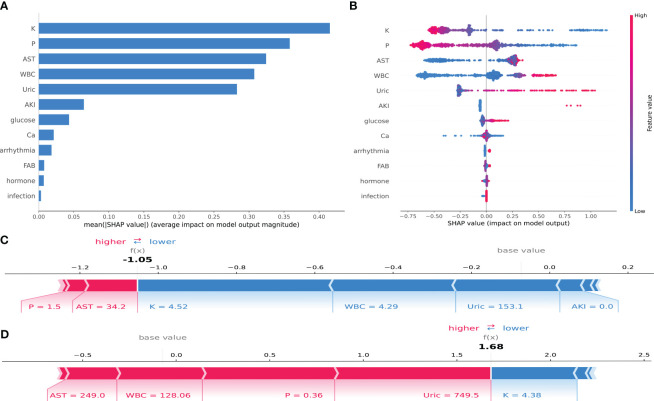
SHAP interpretation of the CatBoost model. **(A)** Importance ranking of features that have an impact on model prediction. **(B)** Impact of each feature on model prediction; in each row, each dot represents a patient, and the color of the dot represents the feature value: red represents a larger value, blue represents a lower value. **(C, D)** Individualized predictions of the model for two patients.

In this study, an analysis was conducted to enhance the interpretability of the CatBoost model by importing the SHAP package into the Tree-Explainer class. **Figure 5A** displays the importance ranking of 12 risk factors evaluated by the average absolute SHAP value, while **Figure 5B** shows the distribution of the impact of each feature on the model output results. The feature ordering (*y*-axis) indicates the importance of the predictors, and the SHAP value (*x*-axis) is a unity index showing the impact of a particular variable on the model output results. Each dot in each row represents a patient, and the color of the dot represents the feature value: red for higher values and blue for lower values. Red and blue bars represent risk factors and protective factors, respectively; the longer bars indicate more significant feature importance. We observe that potassium has the most critical influence, with lower potassium exhibiting a positive SHAP value (dots extending to the right becoming increasingly blue) and higher blood potassium exhibiting a negative SHAP value (dots extending to the left becoming increasingly red). This suggests that when potassium concentrations are very low, it increases the risk of TLS in children. The opposite is true for WBC—the higher the WBC, the higher the predictive value for TLS. The distribution of dots can also provide valuable insights, such as in the case of UA, where we can see a dense cluster of low UA (blue dots) associated with a negative SHAP value. The instances of high uric acid (red dots) extend further to the right, indicating that high UA has a greater positive impact on the occurrence of TLS than low UA.

**Supplementary Figure S1** displays the SHAP scatter dependency plots for the first five characteristics, revealing considerable variation in the relationship between the SHAP values and the values of the variables within each characteristic. Potassium and phosphorus show a negative, nearly linear trend across the range of values. The SHAP values for white blood cell count, uric acid, and glutamic transaminase are comparable over a range of values but then increase sharply. When potassium = 3.68 mmol/L, phosphorus = 1.49 mmol/L, AST = 33.6 U/L, WBC = 16.6*10^9^, and UA = 512 µmol/L, the resulting SHAP value is 0. The greater the SHAP value, the higher the probability of TLS occurrence.

To illustrate the interpretability of the model, we present two typical examples: **Figure 5C** represents a pediatric ALL patient with phosphorus = 1.5 mmol/L, AST = 34.2 U/L, potassium = 4.52 mmol/L, WBC = 4.29*10^9^, and UA = 153.1 mmol/L. The CatBoost model predicts a SHAP value of −1.05 for this patient, with a 25.92% probability of developing TLS. **Figure 5D** represents a pediatric ALL patient with AST = 249.0 U/L, WBC = 128.06*10^9^, phosphorus = 0.36 mmol/L, and potassium = 4.38 mmol/L. Under the prediction of the CatBoost model, this patient has a SHAP value of 1.68 and an 84.29% probability of developing TLS.

## 4 Discussion

This study is the first to use machine learning algorithms to identify risk factors for the development of TLS in pediatric ALL patients and to construct machine learning models to assess the probability of TLS in these patients. Several studies have shown that models based on machine learning algorithms perform better than predictive models using traditional logistic regression. In 2021, Alabi et al. developed a machine-learning model to predict overall survival in tongue cancer, and when compared to traditional logistic regression using an externally validated dataset, the machine-learning model had an accuracy of 88.7%, compared to logistic regression’s accuracy of 60.4% (33). In 2022, Zhao et al. developed a machine learning-based RF model to predict the maximum creatinine value within 24 h after diagnosis of AKI patients as an independent predictor of renal function prognosis, and the RF model showed better ability in predicting the prognosis of patients with AKI compared with traditional regression models (34). Although several studies have examined the risk factors for TLS in children with ALL (35, 36), traditional logistic prediction models based on a single center for the occurrence of TLS in adults with AML and children with ALL have also been established in previous studies abroad, but machine learning-based risk models have not been reported.

TLS is the most common disease-related hematologic cancer-critical illness in children. TLS is most commonly seen in fast-growing and chemotherapy-sensitive malignancies, such as non-Hodgkin’s lymphoma, acute nonlymphoblastic leukemia, and acute lymphoblastic leukemia among hematologic neoplasms, as well as in certain solid tumors of epithelial origin, such as small-cell lung carcinomas, advanced breast carcinomas, and neural tube cell tumors (15). The incidence and prevalence of TLS are not well defined because they depend on tumor type, treatment regimen, patient-related risk factors, and preventive measures taken. Most epidemiologic data, mostly from the 1990s, show that up to 70% of children with acute leukemia present with LTLS (37) and <10% had clinical manifestations of LTLS (19, 38, 39). Early experience has led to rigorous prophylaxis and step-dosing treatment strategies that have significantly reduced the incidence of TLS (40). However, once TLS occurs, the consequences can be fatal. A retrospective study of patients with TLS from 2010 to 2013 found that the majority (58%) of patients developed acute renal failure, often with comorbid infections and sepsis, with a very low rate of seizures (1%), and an overall mortality rate of 21 (13). The most common causes of death in patients with clinical TLS are hemorrhage and renal failure (19, 27, 41). Although TLS poses a challenge to treatment, it is generally curable for most patients with TLS through early detection and the use of appropriately designed prevention programs. Regarding risk factors for developing TLS, known reports include disease type, tumor volume (assessed by solid tumor size, LDH, or white blood cell count), renal insufficiency, and dehydration status (42).

In this study, the AUC of the CatBoost model constructed with 12 predictor variables screened by logistic LASSO regression was 0.803 (95% CI: 0.735–0.865). The factors used to construct the model included FAB typing, white blood cell count at initial diagnosis, blood phosphorus, blood calcium, blood potassium, uric acid, glutamic acid aminotransferase, blood glucose, the occurrence of infections, acute kidney injury, cardiac arrhythmia, and the type of steroid used in the initial induction chemotherapy. Previous studies have shown that TLS occurs most frequently in patients with acute lymphoblastic leukemia type L3, high initial white blood cell counts, high uric acid levels, and renal insufficiency (43, 44), which is the same as the results of our analysis. A few patients with their own high baseline blood phosphorus, calcium, potassium, and uric acid levels were prone to spontaneous tumor lysis syndrome (45). In the present study, we found for the first time that the patients’ glutamine aminotransferase, blood glucose, the occurrence of infection, acute kidney injury, cardiac arrhythmia, and the type of steroid used at the time of the initial induction chemotherapy were associated with the development of TLS at the time of the initial diagnosis.

It is well known that the typical clinical manifestations of TLS are hyperuricemia, hyperkalemia, hyperphosphatemia, and hypocalcemia (25, 46, 47). However, when we used SHAP to interpret the optimal CatBoost model, the results suggested that lower potassium and lower phosphorous were more likely to occur in TLS. On the one hand, the data on potassium and phosphorus we collected were at the time of the diagnosis of ALL, when the patients had not yet started chemotherapy, and a large number of tumor cells sensitive to the response to initial chemotherapy may not have yet cracked in large numbers, and a very small number of patients with spontaneous TLS were outside the scope of our study. On the other hand, patients with initial ALL often present with fever, mouth ulcers, sore throat, chest pain, cough, abdominal pain, diarrhea, skin petechiae and ecchymosis, bleeding gums, nosebleeds, nausea, vomiting, pallor, dizziness, and shortness of breath. At this time, the patients often have poor appetite (48), and the body’s potassium and phosphorus are mainly obtained through plant and animal foods. The most frequent electrolyte abnormality with ALL patients was hypokalemia. Furthermore, hypokalemic patients more frequently experienced concurrent electrolyte disturbances (i.e., hyponatremia, hypocalcemia, hypophosphatemia, and hypomagnesemia) (49). Therefore, low potassium and low phosphorus are consistent with the state of the patients at the time of initial diagnosis.

Arrhythmia refers to an abnormality in the frequency, rhythm, site of origin, conduction velocity, or order of excitation of the heart’s impulses (50). We retrieved all ECG reports for all patients at the time of the initial ALL visit, and the results showed that the incidence of arrhythmia in 2,243 patients was 28.89% (648/2,243), of which the incidence of arrhythmia in patients with TLS was 37.19% (74/199), and the incidence of arrhythmia in patients without TLS was 28.08% (574/2,044). Interestingly, existing studies have found that patients with TLS always have electrolyte and metabolic disturbances, and it can progress to clinical toxic effects, including cardiac arrhythmias due to hyperkalemia and hypocalcemia (51). In our study, a significant proportion of patients with a diagnosis of ALL already had arrhythmias at the time of diagnosis, regardless of whether or not TLS occurred after starting chemotherapy.

However, this study has several limitations. Firstly, the predictive model was constructed based on a retrospective study conducted in a single center in China. Therefore, its predictive validity needs to be further verified using external data, particularly large-sample cohort studies involving multiple centers, different regions, and diverse ethnicities. Secondly, the MissForest algorithm used in this study has a missing value limit of <30% for interpolating mixed missing data into good data. As a result, variables with missing values exceeding 30% (such as BMI and calcitoninogen) were not included in the analysis. Thirdly, the analysis did not consider variables such as mutations in specific genes due to the high cost and low prevalence of genetic testing in the early stages. Despite these limitations, this study represents the first attempt to develop a machine-learning model for predicting the risk of TLS in children with ALL in China.

## 5 Conclusion

This study examined 12 risk factors associated with the development of TLS using data from ALL patients. A risk prediction model was established based on these factors. The study results demonstrated the good predictive ability of the CatBoost model. The CatBoost model can be incorporated into a clinical decision support system to assist clinicians in making effective diagnoses and treatment decisions. This can help prevent or delay the occurrence of TLS in ALL patients, particularly high-risk children, thereby reducing the economic burden on patients and society. Additionally, the use of TLS risk alerts during clinic visits for ALL patients can minimize missed diagnoses.

## Data availability statement

The original contributions presented in the study are included in the article/**Supplementary Material**, further inquiries can be directed to the corresponding author.

## Ethics statement

The studies involving humans were approved by The ethics committee of the Children’s Hospital of Chongqing Medical University. The studies were conducted in accordance with the local legislation and institutional requirements. The ethics committee/institutional review board waived the requirement of written informed consent for participation from the participants or the participants’ legal guardians/next of kin because The project was a retrospective study utilizing medical records obtained from previous clinical consultations, without unnecessary risk to the subjects, and informed consent was agreed to be waived with a commitment not to disclose patient privacy.

## Author contributions

YX: Writing – original draft. LX: Writing – original draft. YZ: Writing – original draft. XX: Writing – review & editing. XG: Writing – review & editing. YG: Writing – review & editing. YS: Writing – review & editing. XL: Writing – review & editing. YD: Writing – review & editing. JY: Writing – review & editing.
