# Supplementary material for: Prediction of tumor lysis syndrome in childhood acute lymphoblastic leukemia based on machine learning models: a retrospective study
Source: Front Oncol. 2024 Mar 7;14:1337295. doi: 10.3389/fonc.2024.1337295 (PMC10955075; doi:10.3389/fonc.2024.1337295)

**Construction of Prediction of Tumor Lysis Syndrome in Childhood Acute Lymphoblastic Leukemia Based on Machine Learning Models: a Retrospective Study**

**Contents**

**Table S1.**...........................................................................................................................................2

**Table S2.**...........................................................................................................................................4

**Figure S1.**.........................................................................................................................................5

**TABLE S1** Demographic and clinical characteristics of study participants

| **Characteristic** | **Overall** | **No TLS** | **TLS** | **P-value** |
| --- | --- | --- | --- | --- |
|  | **N=2243** | **N=2044** | **N=199** |  |
| **Gender, n(%)** |  |  |  | 0.0303 |
| Male | 1332 (59.38) | 1199 (58.66) | 133 (66.83) |  |
| Female | 911 (40.62) | 845 (41.34) | 66 (33.17) |  |
| **Age (year)**  **median**  **IQR** | 4.750  [3.000, 8.250] | 4.830  [3.000, 8.330] | 4.330  [2.670, 7.085] | 0.0542 |
| **immunophenotype, n (%)** |  |  |  |  |
| Common B-cell | 2041 (90.99) | 1871 (91.54) | 170 (85.43) | <0.0001 |
| Precursor B-cell | 107 (4.77) | 100 (4.89) | 7 (3.52) |  |
| T-cell | 89 (3.97) | 68 (3.33) | 21 (10.55) |  |
| Other (biphenotypic) | 6 (0.27) | 5 (0.24) | 1 (0.50) |  |
| **FAB, n (%)** |  |  |  |  |
| L1 | 830 (37.00) | 769 (37.62) | 61 (30.65) | 0.0014 |
| L2 | 1191 (53.10) | 1088 (53.23) | 103 (51.76) |  |
| L3 | 148 (6.60) | 124 (6.07) | 24 (12.06) |  |
| Unclassified | 74 (3.30) | 63 (3.08) | 11 (5.53) |  |
| **regimen, n (%)** |  |  |  |  |
| CCCG-2008 | 758 (33.79) | 703 (34.39) | 55 (27.64) | 0.1551 |
| CCCG-2015 | 1040 (46.37) | 940 (45.99) | 100 (50.25) |  |
| CCCG-2020 | 445 (19.84) | 401 (19.62) | 44 (22.11) |  |
| **WBC****(x10^9^/L)**  **median**  **IQR** | 7.490  [3.490, 26.190] | 6.810  [3.340, 21.407] | 28.480  [7.020, 131.955] | <0.0001 |
| **PLT(x10^9^/L)**  **median**  **IQR** | 57.000  [26.000,131.500] | 59.000  [26.000, 135.000] | 48.000  [26.500, 102.000] | 0.1255 |
| **Hb****(g/L)**  **median**  **IQR** | 81.000  [69.000, 98.000] | 81.000  [69.000, 98.000] | 82.000  [69.000, 97.000] | 0.9145 |
| **Cr****(umol/L)**  **Median**  **IQR** | 32.000  [26.000, 41.100] | 31.750  [25.875, 40.000] | 41.000  [29.000, 53.750] | <0.0001 |
| **Urea****(mmol/L)**  **Median**  **IQR** | 4.300  [3.400, 5.365] | 4.315  [3.420, 5.370] | 4.050  [2.910, 5.265] | 0.0274 |
| **Ca****(mmol/L)**  **Median**  **IQR** | 2.330  [2.220, 2.440] | 2.330  [2.228, 2.440] | 2.300  [2.180, 2.450] | 0.0635 |
| **P(mmol/L)**  **Median**  **IQR** | 1.570  [1.350, 1.760] | 1.590  [1.380, 1.780] | 1.320  [1.050, 1.555] | <0.0001 |
| **K****(mmol/L)**  **median**  **IQR** | 4.120  [3.820, 4.400] | 4.140  [3.860, 4.420] | 3.720  [3.280, 4.110] | <0.0001 |
| **Uric****(umol/L)**  **median**  **IQR** | 323.000 [257.000,434.400] | 318.000  [253.950, 418.925] | 483.000  [303.000, 698.500] | <0.0001 |
| **LDH****(U/L)**  **median**  **IQR** | 421.000  [261.200,906.250] | 392.200  [256.300, 803.150] | 1073.000  [450.900, 2447.800] | <0.0001 |
| **AST(U/L)**  **median**  **IQR** | 34.000  [25.000, 53.900] | 33.300  [24.900, 51.300] | 49.900  [32.650, 94.450] | <0.0001 |
| **ALT(U/L)**  **median**  **IQR** | 22.600  [14.000, 35.900] | 22.300  [14.000, 35.225] | 26.900  [16.350, 46.200] | 0.0006 |
| **TBil(umol/L)**  **median**  **IQR** | 6.800  [4.200, 10.000] | 6.700  [4.200, 9.900] | 7.400  [4.400, 12.100] | 0.0382 |
| **Fib(g/L)**  **median**  **IQR** | 2.760  [2.060, 3.680] | 2.800  [2.100, 3.703] | 2.500  [1.800, 3.340] | 0.0001 |
| **Blasts (%)**  **median**  **IQR** | 0.200  [0.000, 0.650] | 0.170  [0.000, 0.630] | 0.560  [0.040, 0.800] | <0.0001 |
| **Glucose(mmol/L)**  **median**  **IQR** | 5.130  [4.550, 5.810] | 5.110  [4.560, 5.782] | 5.370  [4.430, 6.350] | 0.0866 |
| **PT(s)**  **median**  **IQR** | 12.100  [11.300, 13.100] | 12.000  [11.200, 13.000] | 12.700  [11.800, 14.300] | <0.0001 |
| **CRP(mg/L)**  **median**  **IQR** | 12.000  [8.000, 21.215] | 12.000  [8.000, 21.260] | 12.000  [8.000, 20.630] | 0.7581 |
| **hemorrhage, n (%)** |  |  |  | 0.0014 |
| No | 1217 (54.26) | 1131 (55.33) | 86 (43.22) |  |
| Yes | 1026 (45.74) | 913 (44.67) | 113 (56.78) |  |
| **epilepsy, n (%)** |  |  |  | 0.666 |
| No | 2233 (99.55) | 2034 (99.51) | 199 (100.00) |  |
| Yes | 10 (0.45) | 10 (0.49) | 0 (0.00) |  |
| **convulsions, n (%)** |  |  |  | 0.7345 |
| No | 2230 (99.42) | 2033 (99.46) | 197 (98.99) |  |
| Yes | 13 (0.58) | 11 (0.54) | 2 (1.01) |  |
| **infection, n (%)** |  |  |  | 0.3183 |
| No | 157 (7.00) | 147 (7.19) | 10 (5.03) |  |
| Yes | 2086 (93.00) | 1897 (92.81) | 189 (94.97) |  |
| **HF, n (%)** |  |  |  | 0.0722 |
| No | 2233 (99.55) | 2037 (99.66) | 196 (98.49) |  |
| Yes | 10 (0.45) | 7 (0.34) | 3 (1.51) |  |
| **AKI, n (%)** |  |  |  | <0.0001 |
| No | 2216 (98.80) | 2039 (99.76) | 177 (88.94) |  |
| Yes | 27 (1.20) | 5 (0.24) | 22 (11.06) |  |
| **arrhythmia, n (%)** |  |  |  | 0.0087 |
| No | 1595 (71.11) | 1470 (71.92) | 125 (62.81) |  |
| Yes | 648 (28.89) | 574 (28.08) | 74 (37.19) |  |
| **hepatomegaly, n (%)** |  |  |  | 0.0772 |
| No | 476 (21.22) | 444 (21.72) | 32 (16.08) |  |
| Yes | 1767 (78.78) | 1600 (78.28) | 167 (83.92) |  |
| **splenomegaly, n (%)** |  |  |  | 0.0035 |
| No | 1027 (45.79) | 956 (46.77) | 71 (35.68) |  |
| Yes | 1216 (54.21) | 1088 (53.23) | 128 (64.32) |  |
| **Steroid, n (%)** |  |  |  | 0.2658 |
| Pred | 483 (21.53) | 447 (21.87) | 36 (18.09) |  |
| Dex | 1614 (71.96) | 1468 (71.82) | 146 (73.37) |  |
| Unknown | 146 (6.51) | 129 (6.31) | 17 (8.54) |  |

Abbreviations: *FAB* French–American–British classification systems;*WBC* white blood cell count;*Hb* hemoglobin;*P* phosphorus;*K* potassium;*Uric* uric acid;*Cr* creatininer*;LDH lactate dehydrogenase*;*AST* aspartate transaminase ;*ALT* alanine transaminase;*TBil* total bilirubin;*Fib* fibrinogen ;*Blasts* primitive immature cells;*PT* prothrombin time ;*CRP* C-reactive protein;*HF* heart failure ;*AKI* acute kidney injury;*Pred* prednisone;*Dex* dexamethasone

**TABLE S2** Optimal parameters of the model.

| mould | Selection of parameters | optimal parameter |
| --- | --- | --- |
| RF | n_estimators: [100, 200, 400, 800, 1000,1200],  min_samples_split: range(2, 22, 1), max_features: range(5, 35, 1), max_features: range(5, 35, 1)  max_features: range(5, 35, 1)  max_depth: range(1, 22, 1) | min_samples_split=11,  n_estimators=100,  max_features=5, max_depth=3  max_depth=3 |
| logistic | penalty: ['l1', 'l2'],  C: [0.005, 0.05, 0.5, 5,10],  solver: ['lbfgs', 'liblinear'] | C=5,  penalty='l1',  solver='liblinear' |
| CatBoost | depth:range(1, 10, 1),  learning_rate:[0.01,0.03,0.05,0.1],  l2_leaf_reg:range(1, 5, 1),  iterations:[100,300,500,1000] | depth=2,  learning_rate=0.01,  l2_leaf_reg=4,  iterations=500 |
| SVM | kernel: ['rbf', 'linear', 'poly'],  C: [0.001, 0.01, 1, 10, 100],  gamma: [0.001, 0.01, 1, 10, 100] | kernel='linear',  C=100,  gamma=0.5 |

**Figure S1** SHAP dependence plots for the top 5 clinical features contributing to the prediction of the CatBoost model. (A) Potassium (B) Phosphorus (C) AST (D) WBC (E) Uric acid .


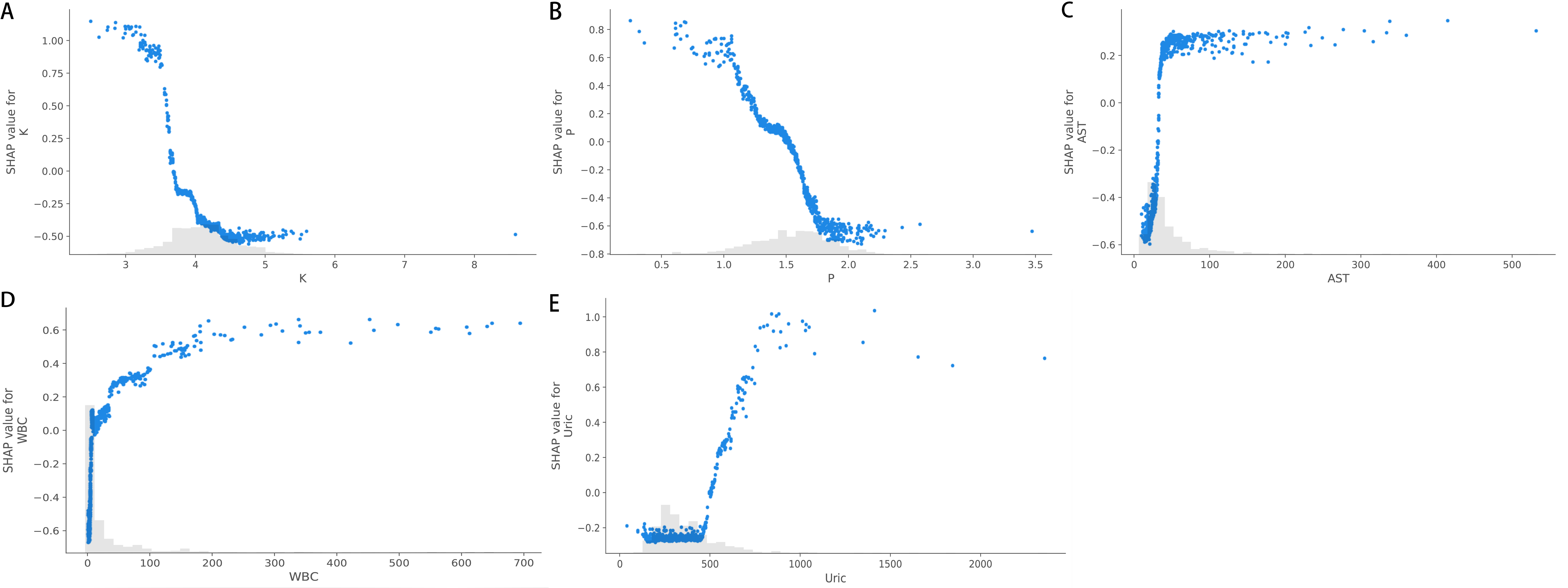

Supplement: Supplementary file 1 [file DataSheet_1.docx]
